# Managing hyperglycemia and rash associated with alpelisib: expert consensus recommendations using the Delphi technique

**DOI:** 10.1038/s41523-024-00613-x

**Published:** 2024-01-31

**Authors:** Emily J. Gallagher, Heather Moore, Mario E. Lacouture, Susan F. Dent, Azeez Farooki, Marcus D. Goncalves, Claudine Isaacs, Abigail Johnston, Dejan Juric, Zoe Quandt, Laura Spring, Brian Berman, Melanie Decker, Gabriel N. Hortobagyi, Benjamin H. Kaffenberger, Bernice Y. Kwong, Timothy Pluard, Ruta Rao, Lee Schwartzberg, Michael S. Broder

**Affiliations:** 1 Division of Endocrinology, Diabetes and Bone Disease, Department of Medicine, and Tisch Cancer Institute, Icahn School of Medicine at Mount Sinai, New York, NY USA; 2 Duke Cancer Institute, Duke University, Durham, NC USA; 3 Department of Medicine, Memorial Sloan Kettering Cancer Center, New York, NY USA; 4 Division of Endocrinology, Weill Department of Medicine, Weill Cornell Medicine, New York, NY USA; 5 Lombardi Comprehensive Cancer Center, Georgetown University, Washington, DC USA; 6 Surviving Breast Cancer, 305 Pink Pack, Miami, FL USA; 7 Massachusetts General Hospital Cancer Center, Department of Medicine, Harvard Medical School, Boston, MA USA; 8 School of Medicine, University of California, San Francisco, CA USA; 9 University of Miami School of Medicine and Center for Clinical and Cosmetic Research, Aventura, FL USA; 10 Woodland Memorial Hospital, Woodland, CA, and Kaiser Permanente, Sacramento, CA USA; 11 Department of Breast Medical Oncology, The University of Texas MD Anderson Cancer Center, Houston, TX USA; 12 Wexner Medical Center, The Ohio State University, Columbus, OH USA; 13 Department of Dermatology, Stanford University School of Medicine, Stanford, CA USA; 14 St. Luke’s Hospital Koontz Center for Advanced Breast Cancer, Kansas City, MO USA; 15 Rush Hematology, Oncology and Cell Therapy, Rush University Medical Center, Chicago, IL USA; 16 West Cancer Center, Memphis, TN USA; 17 PHAR, Beverly Hills, CA USA; 18 Present Address: Division of Endocrinology, Weill Department of Medicine, Weill Cornell Medicine, New York, NY USA

**Keywords:** Cancer, Breast cancer

## Abstract

Hyperglycemia and rash are expected but challenging adverse events of phosphatidylinositol-3-kinase inhibition (such as with alpelisib). Two modified Delphi panels were conducted to provide consensus recommendations for managing hyperglycemia and rash in patients taking alpelisib. Experts rated the appropriateness of interventions on a 1-to-9 scale; median scores and dispersion were used to classify the levels of agreement. Per the hyperglycemia panel, it is appropriate to start alpelisib in patients with HbA1c 6.5% (diabetes) to <8%, or at highest risk for developing hyperglycemia, if they have a pre-treatment endocrinology consult. Recommend prophylactic metformin in patients with baseline HbA1c 5.7% to 6.4%. Metformin is the preferred first-line anti-hyperglycemic agent. Per the rash panel, initiate prophylactic nonsedating H1 antihistamines in patients starting alpelisib. Nonsedating H1 antihistamines and topical steroids are the preferred initial management for rash. In addition to clinical trial evidence, these recommendations will help address gaps encountered in clinical practice.

## Introduction

Phosphatidylinositol-4,5-bisphosphate 3-kinase catalytic subunit alpha (*PIK3CA*) is the gene that encodes the p110α catalytic subunit of phosphatidylinositol-3-kinase (PI3K) class IA and is the most frequently mutated gene of the PI3K pathway in cancer^1–3^. Approximately 40% of patients with hormone receptor-positive (HR+), human epidermal growth factor receptor 2-negative (HER2–) advanced breast cancer (ABC) have a *PIK3CA* mutation^4–8^. *PIK3CA* oncogenic driver mutations can cause PI3K pathway hyperactivation, which can lead to tumor growth and contribute to endocrine resistance in HR+, HER2– ABC^9–12^. Alpelisib is an α-selective PI3K inhibitor (PI3Ki) and degrader of mutant p110α, approved in combination with fulvestrant for the treatment of postmenopausal women and men with *PIK3CA*-mutated HR+, HER2– ABC after progression on endocrine therapy (ET), based on the results of the Phase III SOLAR-1 trial^13–17^. In SOLAR-1, patients with *PIK3CA*-mutated HR+, HER2– ABC treated with alpelisib and fulvestrant (*n* = 169) had significantly longer progression-free survival (PFS) compared with those who received placebo and fulvestrant (*n* = 172; median PFS 11 months vs 5.7 months, hazard ratio 0.65, *P* < 0.001)^17^. Approximately 30% (*n* = 51) of patients with *PIK3CA*-mutated disease treated with alpelisib had long-term disease control (PFS ≥ 18 months), and 72.5% of these patients had PFS ≥ 24 months^18^.

In SOLAR-1, the most common any-grade adverse events (AEs) observed with alpelisib (*N* = 284) were hyperglycemia (63.7%), diarrhea (57.7%), nausea (44.7%), decreased appetite (35.6%), and rash (35.6%). Hyperglycemia (6.3%) and rash (3.2%) were the most common AEs leading to alpelisib discontinuation^17^. Median time to first onset of grade ≥2 hyperglycemia (fasting glucose >160 mg/dL or >8.9 mmol/L) was 15 days and 12 days for grade 2–3 rash (≥10% body surface area (BSA) with active skin toxicity; no grade 4 rash was reported in SOLAR-1)^15,19^. A similar safety profile of alpelisib was observed in the Phase II 3-cohort BYLieve study, which assesses the safety and efficacy of alpelisib with ET (fulvestrant/letrozole) in patients with HR+, HER2–, *PIK3CA*-mutated ABC whose disease progressed on/after prior treatments^17,20–23^.

Hyperglycemia and rash may be considered expected AEs of alpelisib resulting from inhibition of the PI3K pathway, which controls various cellular and physiologic functions^24,25^. For instance, p110α plays a key role in glucose metabolism, because it mediates the response of skeletal muscle, liver, and fat to insulin. Inhibition of PI3K leads to acute insulin resistance, blocking glucose uptake in skeletal muscle and adipose tissue, activating hepatic glycogenolysis, causing hyperglycemia and a compensatory increase in circulating insulin^1,26,27^. Preclinical data suggest that the resulting hyperinsulinemia can partially reactivate the PI3K pathway^26^. Acute hyperglycemia needs to be diagnosed and acted upon because it can cause volume depletion, electrolyte disturbances, catabolic weight loss, and, very rarely, hyperosmolar state or diabetic ketoacidosis^28^. The PI3K pathway is also involved in skin homeostasis, with a crucial role in maintaining the epidermal barrier, hair follicle regeneration, dermal wound healing, and skin senescence^29^. Histamine-producing cells and eosinophils may play a role in rash development in patients taking alpelisib. Rash associated with alpelisib is frequently maculopapular and/or pruritic and has histologic characteristics consistent with a hypersensitivity reaction^30^.

Current guidance for the management of hyperglycemia and rash in patients receiving alpelisib is primarily based on clinical trial experience, which is not necessarily reflective of the experience in real-world patients. SOLAR-1 did not include comprehensive prospective guidance on AE management. Detailed guidance is lacking on certain aspects of AE management, such as which anti-hyperglycemic agent should be used in the first and subsequent lines for blood glucose elevations. Hence, management of these AEs remains a challenge. In the absence of definitive evidence, expert consensus recommendations can provide clinically useful guidance for AE management. The objective of this study is to provide practical recommendations for optimizing prevention and management of hyperglycemia and rash in patients receiving alpelisib based on an integrated Delphi panel.

## Results

### Hyperglycemia treatment and management consensus recommendations per areas of agreement

Experts reviewed 624 scenarios for round 1 and 525 scenarios for round 2. Agreement was reached in 83% of ratings for round 2. In the follow-up panel, experts reviewed 284 scenarios and reached agreement in 96% of ratings.

Based on expert consensus, patients who are ≥70 years old, have obesity (body mass index (BMI) ≥ 30 kg/m^2^), and glycosylated hemoglobin (HbA1c) 5.7–6.4% are considered at highest risk for developing new-onset hyperglycemia, and it is recommended to refer these patients for endocrinology evaluation before initiating alpelisib. It is generally unnecessary to refer the lowest-risk patients (no obesity, HbA1c < 5.7%) for an endocrinology evaluation. For all patients with type 2 diabetes mellitus and/or HbA1c 6.5% to <8.0%, it is inappropriate to consider alpelisib treatment without a pre-treatment endocrinology consultation. Providers can consider initiating prophylactic metformin therapy (dose escalate as needed up to 2000 or 2500 mg/day if glomerular filtration rate (GFR) is >45 mL/minute/1.73 m^2^) with or without a second-line agent, such as a sodium-glucose co-transporter 2 inhibitor (SGLT2i) or a thiazolidinedione (TZD), for high-risk patients waiting for endocrinology evaluation.

The panel agreed that prior to starting alpelisib, it is appropriate to recommend a low-carbohydrate diet (60–130 g/day) in all patients and consult a dietician as needed; it may also be appropriate to recommend a ketogenic diet (total carbohydrate intake of <50 g/day) and/or pre-treatment fasting (e.g., >12 hours of food restriction prior to dosing alpelisib daily). Prophylactic metformin (short-acting or extended-release) is recommended for all patients with baseline HbA1c of 5.7–6.4%, and it may be appropriate for patients with HbA1c < 5.7%. In patients at highest risk for developing hyperglycemia, there was disagreement on recommending prophylactic metformin and a second anti-hyperglycemic agent.

For most patients on alpelisib, the panel recommended weekly fasting blood glucose (FBG) monitoring, which can be done using a point-of-care glucose monitor. For patients with intermediate risk of developing hyperglycemia (obesity and HbA1c 5.7–6.4%), twice-weekly FBG monitoring is preferred. For patients at highest risk for developing hyperglycemia, daily FBG monitoring is recommended; daily and twice-weekly monitoring can also be done with an at-home glucose monitor. If persistent hyperglycemia develops, testing blood glucose twice daily may be considered, once before breakfast with ≥8 hours of fasting prior to testing and once before dinner without fasting.

For patients who developed hyperglycemia while on alpelisib, metformin (short-acting or extended-release) is the preferred first-line anti-hyperglycemic agent. Metformin may be increased up to 2000 mg/day (provided a GFR of >45 mL/minute/1.73 m^2^), or up to 2500 mg/day. However, experts suggested that the efficacy of 2500 mg/day may not be improved over 2000 mg/day. Either an SGLT2i or a TZD is an appropriate second- or third-line agent, or first-line therapy in metformin-intolerant patients. Glucagon-like peptide 1 receptor agonists may also be appropriate in these settings if the patient is not experiencing significant gastrointestinal side effects or weight loss. Insulin, sulfonylureas, and dipeptidyl peptidase-4 inhibitors (DPP4i) are generally not appropriate first- or second-line agents. A DPP4i may be an appropriate third-line agent. Experts did not recommend requiring ketone monitoring while on SGLT2i therapy (SGLT2is carry a risk of euglycemic diabetic ketoacidosis), but this monitoring can be done per provider discretion^28,31^.

**Fig. 1:**
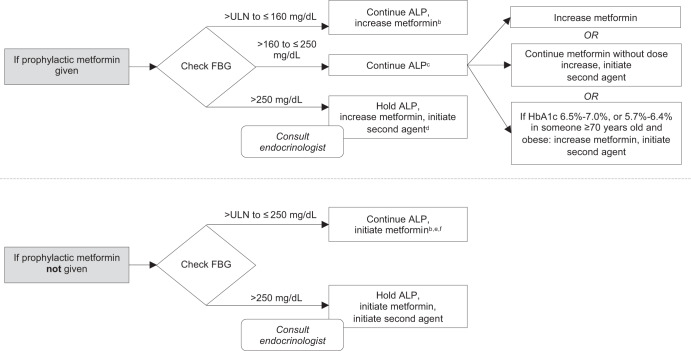
Consensus treatment algorithm for the management of the first episode of hyperglycemia associated with alpelisib. ALP alpelisib, FBG fasting blood glucose, HbA1c glycosylated hemoglobin, MTD maximum-tolerated dose, ULN upper limit of normal. ^a^Unless otherwise stated for all statements about increasing metformin, assume extended-release or short-acting, and up to MTD. ^b^In certain circumstances (e.g., select patients who continue to have HbA1c < 8.0% or those who are asymptomatic and intolerant to metformin), it may be appropriate to continue ALP without initiating or changing metformin dose. ^c^It may also be appropriate to temporarily hold ALP (with the intent to restart at same dose) and increase metformin in certain high-risk patients (e.g., HbA1c > 5.7%). ^d^If FBG > 250 to ≤500 mg/dL, it may also be appropriate to hold or dose reduce ALP without first holding and continue metformin without a dose increase (metformin not at MTD) while simultaneously initiating a second agent. ^e^With the goal of titrating to maximum dose of 2000 mg/day within 1 week^32^. ^f^If FBG > ULN to ≤250 mg/dL, it may also be appropriate to either (1) continue ALP while simultaneously initiating metformin and a second agent or, (2) hold ALP while simultaneously initiating metformin and a second agent in certain high-risk patients (e.g., HbA1c ≥ 6.5%).

**Fig. 2:**
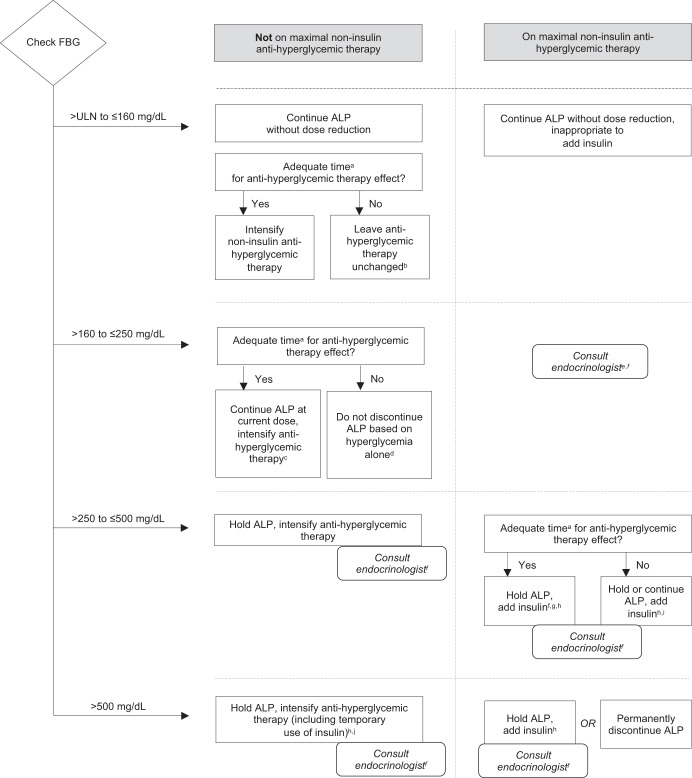
Consensus treatment algorithm for the management of subsequent episodes of hyperglycemia associated with alpelisib. ALP alpelisib, DPP4i dipeptidyl peptidase-4 inhibitor, FBG fasting blood glucose, GLP-1 RA glucagon-like peptide 1 receptor agonist, HbA1c glycosylated hemoglobin, SGLT2i sodium-glucose co-transporter 2 inhibitor, TZDs thiazolidinediones, ULN upper limit of normal. ^a^Metformin: 2 weeks, SGLT2i: 2 days, DPP4i: 1 week, TZDs: 6 weeks, GLP-1 RA: 1 week. ^b^It may also be appropriate to intensify non-insulin anti-hyperglycemic therapy depending on the specific circumstances. ^c^It may be appropriate to consult an endocrinologist to assist with intensifying anti-hyperglycemic treatments. It may also be appropriate to temporarily hold ALP and intensify anti-hyperglycemic treatment. ^d^It may be appropriate to continue ALP either with or without intensifying anti-hyperglycemic therapy, or to temporarily hold ALP and intensify anti-hyperglycemic therapy. ^e^It may be appropriate to give insulin, depending on individual circumstances. It may also be appropriate to either continue or hold ALP. ^f^Or have the patient evaluated in the emergency department if circumstances warrant it. ^g^It may also be appropriate to continue ALP and add standing insulin. ^h^Insulin may reverse catabolic weight loss caused by sustained hyperglycemia. Exercise caution on the use of insulin when holding ALP. Holding ALP may likely cause hyperglycemia to resolve, and adding insulin may lead to hypoglycemia. ^i^Depending upon individual patient circumstances. Insulin can achieve rapid control of hyperglycemia but carries the potential risk of PI3K pathway stimulation^26^. ^j^It may also be appropriate to permanently discontinue ALP depending on the patient’s clinical status.

In certain instances, patients may require combination anti-hyperglycemic therapy and/or holding alpelisib to manage hyperglycemia. For patients with FBG > 250 mg/dL (first episode), the panel recommended holding alpelisib, adding another anti-hyperglycemic agent to metformin, and an endocrinology consult. For patients with FBG > 500 mg/dL at second or later episodes who are on maximal non-insulin anti-hyperglycemic therapy, the panel recommended holding alpelisib, starting insulin, and an endocrinology consult, or permanent discontinuation of alpelisib. The hyperglycemia consensus treatment algorithms for managing the first and subsequent episodes of hyperglycemia as recommended by the expert panel are included in Fig. 1^32^ and Fig. 2.

In patients who: have had ≥1 dose reduction(s) for hyperglycemia, are currently on 200 mg alpelisib, and have had no FBG elevations (<160 mg/dL) with current anti-hyperglycemic therapy for ≥5 weeks, it is appropriate to increase alpelisib (it may also be appropriate for those who have had no FBG elevations for 3-4 weeks with current anti-hyperglycemic therapy). In patients who: have had ≥1 dose reduction(s) for hyperglycemia, are currently on 250 mg alpelisib, and have had no FBG elevations for ≥3 weeks with current anti-hyperglycemic therapy, it may be appropriate to increase alpelisib or keep at current dose.

It may be appropriate to refer patients who received prophylactic metformin and have FBG > 160 to ≤250 mg/dL at first episode for an endocrinology consult, but there was disagreement regarding this recommendation due to concerns about the potential lack of access to endocrinology care. Among patients who received prophylactic metformin and developed hyperglycemia with FBG > 500 mg/dL at first episode, the panel agreed that it may be appropriate to hold alpelisib and increase metformin without initiating a second agent; however, there was concern that this approach is not a strong enough intervention. There was disagreement among panelists for holding alpelisib in patients with diabetes at baseline who have FBG > 160–250 mg/dL at first episode of hyperglycemia or reducing the dose of alpelisib before first holding for patients with FBG > 250 mg/dL. Areas of disagreement are included in Table 1^33^.

**Table 1:** Areas of **disagreement** in the hyperglycemia and rash panel.

Hyperglycemia panel	Rash panel
• Need for a pre-treatment endocrinology consult prior to starting alpelisib in patients with: ∘ BMI < 30 kg/m^2^ and HbA1c 5.7–6.4%, regardless of age ∘ BMI ≥ 30 kg/m^2^ and HbA1c < 5.7%, regardless of age	• Need for a dermatology consultation for patients experiencing rash covering <10% BSA
• Prophylaxis with metformin and a second anti-hyperglycemic agent in patients: ∘ with BMI ≥ 30 kg/m^2^ and HbA1c 5.7–6.4%, regardless of age ∘ ≥70 years old with BMI ≥ 30 kg/m^2^ and HbA1c < 5.7%	• Using fexofenadine at a dose >360 mg/day or loratadine >20 mg/day to control the rash
• Need for endocrinology consult and/or evaluation in the emergency department in patients who did not receive prophylactic metformin and experienced an initial episode of hyperglycemia at FBG > 160–250 mg/dL, with baseline: ∘ HbA1c ≤ 6.4%, regardless of age and BMI ∘ HbA1c 6.5–7%, BMI < 30 kg/m^2^ and age <70 years	• Administering topical corticosteroids during initial management in patients presenting with any % BSA rash and angioedema
• Need for endocrinology consult and/or evaluation in the emergency department in patients who experienced a second or later episode of hyperglycemia at FBG > ULN-160 mg/dL while on alpelisib 200 or 250 mg/day, not on maximal non-insulin anti-hyperglycemic therapy but adequate time has passed since the last anti-hyperglycemic dose change for the agent to take effect	• Initiating OCS in patients with FBG ≥ 160 mg/dL, who present for the first time with rash covering (a) 10–30% BSA or (b) any % BSA limiting instrumental ADLs
	• Need for a specialist consult and/or hospital admission in patients: ∘ whose treatment with alpelisib was put on hold, with/without OCS therapy, and with rash covering <10% upon re-evaluation ∘ whose treatment with alpelisib was put on hold, with/without OCS therapy, whose Grade 2^a^ rash has improved, and who have FBG < 160 mg/dL

*ADLs* activities of daily living, *BMI* body mass index, *BSA* body surface area, *FBG* fasting blood glucose, *HbA1c* glycosylated hemoglobin, *OCS* oral corticosteroids, *ULN* upper limit of normal.

^a^Includes (1) rash covering 10–30% BSA with or without symptoms or, (2) rash covering >30% BSA with or without mild symptoms or, (3) any % BSA limiting instrumental ADLs (e.g., preparing meals, shopping for groceries or clothes, using the telephone, managing money, etc). Descriptors are consistent with CTCAE v5.0^33^.

Recommendations for alpelisib treatment eligibility of patients with HbA1c ≥ 8.0% at baseline were excluded. Although the experts rated considering alpelisib treatment for patients with HbA1c ≥ 8.0% with a pre-treatment endocrinology consultation with a median score of 4 (“may or may not be inappropriate”) without disagreement, upon further review, they agreed more evidence is needed regarding the use of alpelisib in these patients. However, experts agreed that it is inappropriate to consider treating these patients with alpelisib without a pre-treatment endocrinology consultation.

### Rash treatment and management consensus recommendations per areas of agreement

Experts reviewed 364 scenarios/round for rounds 1 and 2. In round 2, agreement was reached in 79% of scenarios.

Based on expert consensus, it is recommended to give prophylactic nonsedating (H1) antihistamine therapy at standard dose for all patients starting alpelisib. These include once-daily dosing of cetirizine 10 mg, levocetirizine 5 mg, loratadine 10 mg, or fexofenadine 180 mg. Initial management for rash in patients who are on alpelisib include nonsedating H1 antihistamines and topical steroids (class I–III) such as fluocinonide 0.1%, triamcinolone acetonide 0.5%, or betamethasone dipropionate 0.05%. The choice of topical steroids depends on the body part and the affected BSA. Class I–II steroids are typically used on areas with thicker skin (e.g., soles of the feet), and should not be used on the face, groin, or axilla^34^. Nonsedating H1 antihistamines may be escalated if response to standard-dose nonsedating H1 antihistamine is inadequate up to: cetirizine 40 mg/day, levocetirizine 15 mg/day, loratadine 20 mg/day, or fexofenadine 360 mg/day. High doses of nonsedating H1 antihistamines are well tolerated, but some patients may experience somnolence, sedation, or anticholinergic side effects^35–38^. For rash affecting >10% BSA, it is appropriate to consult a specialist (dermatologist or allergist). Oral corticosteroid (OCS) therapy (e.g., prednisone) is recommended in patients with rash affecting ≥10% BSA, with a dose of 0.5–1 mg/kg. However, there was disagreement on recommending the use of OCS for patients with an FBG of ≥160 mg/dL presenting with rash for the first time, affecting 10–30% BSA or any BSA limiting instrumental activities of daily living (ADLs). OCS can lead to, or exacerbate, hyperglycemia^39^; hence, exercise caution on its use in patients with elevated FBG. OCS therapy is generally discontinued within 2 weeks^40^.

Evaluation of rash to determine appropriate management should not only depend on BSA affected, but also its effect on the patient’s ADLs. The only way to ascertain impact on ADLs is by asking the patient or caregiver. Regardless of initial management, re-evaluate patients who developed rash after 1–2 weeks, adjust antihistamine dose and topical steroids as needed.

**Fig. 3:**
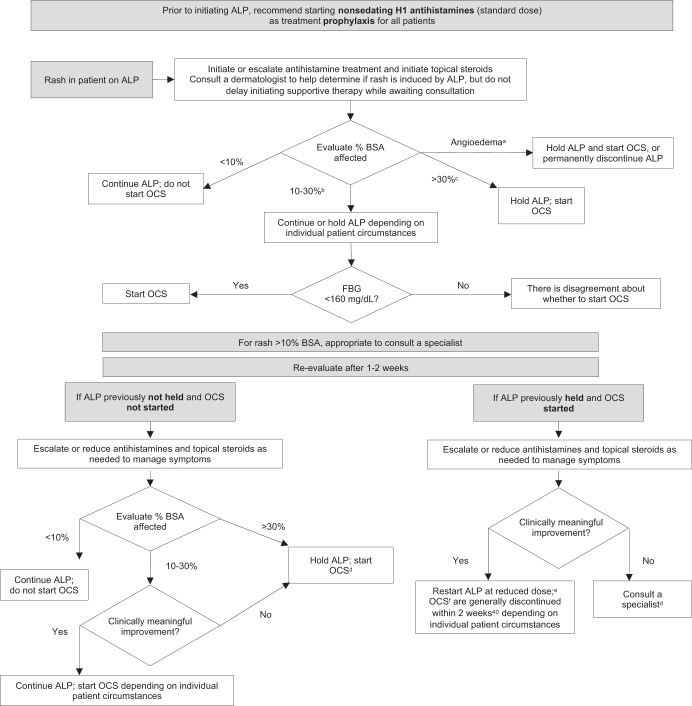
Consensus treatment algorithm for managing rash associated with alpelisib^42^. ADL activities of daily living, ALP alpelisib, BSA body surface area, CTCAE Common Terminology Criteria for Adverse Events, FBG fasting blood glucose, OCS oral corticosteroids. ^a^If angioedema persists or reoccurs, it is appropriate to permanently discontinue ALP and consult a specialist (which can include a dermatologist or allergist) or seek hospital admission for severe or systemic symptoms. ^b^Or if it covers >30% BSA but produces only mild symptoms, or if it limits instrumental ADLs (e.g., preparing meals, shopping for groceries or clothes, using the telephone, managing money, etc) regardless of BSA affected. Descriptors are consistent with CTCAE v5.0^33^. ^c^With moderate or severe symptoms, or if it limits self-care ADLs (e.g., bathing, dressing, and undressing, feeding self, using the toilet, taking medications, and not bedridden) regardless of BSA affected. ^d^Consult a specialist (such as a dermatologist or allergist) or hospital admission for severe or systemic symptoms. ^e^First dose reduction to 250 mg and the second dose reduction to 200 mg. No further dose reductions typically considered. ^f^For patients receiving the prednisone equivalent of ≥20 mg daily for ≥4 weeks, consider prophylaxis against *Pneumocystis jirovecii* pneumonia^43,44^.

**Fig. 4:**
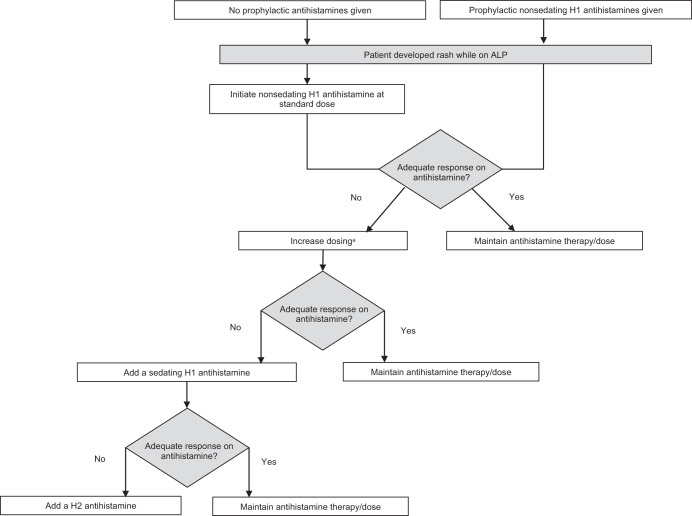
Consensus recommendations for the use of antihistamines to manage rash in patients receiving alpelisib^42^. ALP alpelisib. ^a^Adding a sedating H1 antihistamine to standard-dose nonsedating H1 antihistamine is also appropriate, but escalating nonsedating H1 antihistamines is preferred over adding sedating antihistamines.

Angioedema is an AE associated with alpelisib reported in the postmarketing setting, and refers to the swelling of the deeper layers of the skin, frequently observed in the eyelids, mouth or genitals, typically associated with urticaria or hives^15,41^. Angioedema should be documented by an allergist and/or dermatologist. If a patient receiving alpelisib develops angioedema at initial rash management, either hold alpelisib and start OCS, or permanently discontinue alpelisib. If it persists or reoccurs, permanently discontinue alpelisib. The rash treatment algorithms and recommendations for the use of antihistamines based on the rash expert panel consensus are included in Fig. 3^33,40,42–44^ and Fig. 4^42^.

## Discussion

This study provided expert consensus recommendations for the prevention and management of hyperglycemia and rash, which are two of the most common AEs associated with alpelisib^17^. Optimizing prevention and management strategies for AEs associated with alpelisib treatment may help preserve or improve quality of life, prevent morbidity and early discontinuations due to AEs. Hyperglycemia and rash management involve appropriate patient selection, prophylactic therapy, medical therapy, alpelisib dose modification or discontinuation as necessary, and close monitoring. In patients with elevated fasting plasma glucose (FPG) who discontinued alpelisib in SOLAR-1, FPG levels returned to baseline in 96%, indicating that hyperglycemia associated with alpelisib is reversible^15^.

Other published studies also support the use of prophylactic therapy to prevent hyperglycemia and rash. In the multi-center, prospective Phase II METALLICA trial (baseline glycemic status: normal, *n* = 48; prediabetes, *n* = 20), initiating prophylactic metformin at 500 mg twice daily for days 1–3, and 1000 mg twice daily thereafter prior to alpelisib reduced the incidence of all-grade (METALLICA 42.6%, SOLAR-1 63.7%, BYLieve Cohort A 58.3%) and grade 3-4 (METALLICA 5.9%, SOLAR-1 36.6%, BYLieve Cohort A 28.3%) hyperglycemia; no study discontinuation was due to hyperglycemia^45^. In a single-center retrospective study (baseline glycemic status: normal, *n* = 8; prediabetes, *n* = 5; diabetes, *n* = 2; history of gestational diabetes, *n* = 1) patients treated with prophylactic metformin, TZDs, or SGLT2is prior to initiating alpelisib had fewer incidences of grade 3-4 hyperglycemia than in SOLAR-1^17,46^. In another study of patients on PI3K or protein kinase B (AKT) inhibitors, SGLT2is were associated with the greatest reductions in blood sugar, followed by metformin^47^. In SOLAR-1, prophylactic therapy for rash resulted in lower incidences and reduced severity of rash: incidence of all-grade rash was 27% vs 64%, and grade 3 rash was 12% vs 23% in those who received prophylactic therapy compared with those who did not. A SOLAR-1 protocol amendment was implemented to exclude patients with uncontrolled diabetes, improve AE monitoring, and management after 56.6% of planned patients were enrolled. This resulted in fewer discontinuations due to hyperglycemia (3.6% vs 9%) and any grade (20.7% vs 29.2%) and grade ≥3 (7.9% vs 18.1%) AEs in the second vs the first half of randomized patients. In addition, relatively longer median PFS was observed in patients with *PIK3CA*-mutated tumors who received a higher (≥248 mg/day) than lower (<248 mg/day) median dose intensity of alpelisib, but benefit was still observed compared with those who received placebo^19^. This supports the importance of detailed AE management guidance and keeping patients on the highest tolerated alpelisib therapy.

Most of the available AE management guidance is based on alpelisib clinical trials that have stringent exclusion criteria for baseline characteristics, such as risk factors for developing hyperglycemia, and hence limited in their application in real world. For instance, patients with type 1 and uncontrolled type 2 diabetes (FPG > 140 mg/dL [7.7 mmol/L] or HbA1c of >6.4%) were excluded from SOLAR-1, so the safety of alpelisib in these patients has not been established^15,17^. Most patients in SOLAR-1 and BYLieve received alpelisib in the first to third line, whereas real-world data demonstrate that most patients received alpelisib in fourth line and beyond^17,21–23,48–51^. Furthermore, more patients with Eastern Cooperative Oncology Group performance scores (ECOG PS) ≥ 2 received alpelisib in the real-world (9%) than in SOLAR-1 (ECOG PS 0-1 only) or BYLieve (1.6–3.2%)^17,21–23,51^.

Although expert consensus was reached in multiple areas of AE management in this study as expected, consensus was not reached in others. These include the need for pre-treatment endocrinology consults in patients with certain risk factors for developing hyperglycemia, and use of topical corticosteroids for angioedema during initial rash management. These areas of disagreement emphasize the need for further studies and evidence to guide clinical decision making. In addition, responses that fall within the median score of 4–6 without disagreement may be described as either may or may not be appropriate. However, the experts’ review of aggregated data and final recommendations were key to ensure that this methodological observation did not impact the clinical implications of each recommendation. Although these recommendations can be used to guide alpelisib AE management, further studies are needed to establish their effect on patient outcomes. The ongoing Targeting Insulin Feedback to Enhance Alpelisib (TIFA) trial (NCT05090358) is examining the effects of ketogenic diet, low-carbohydrate diet, or SGLT2i therapy in preventing hyperglycemia in patients with HR+, HER2–, *PIK3CA*-mutated ABC treated with alpelisib^52^.

Delphi panel studies are designed to minimize biases resulting from dominant participants, communication noise, and peer pressure for conformity typically encountered in face-to-face discussions. This is accomplished by following 3 distinct characteristics: anonymity, iteration and controlled feedback, and statistical group response^53^. A limitation of this study is the composition of experts included in each panel, as most of the experts are based in academic institutions and none of the experts in the hyperglycemia panel worked in a community or non-academic setting. In addition, all experts are currently in clinical practice in the United States.

Consensus recommendations provided in this study are based on expert recommendations and their clinical experience. Until data from new clinical trial and real-world studies become available, these expert recommendations can provide practical guidance on AE management for patients treated with alpelisib in clinical practice. A plain language summary of this study is included in the Supplementary Information.

## Methods

**Fig. 5:**
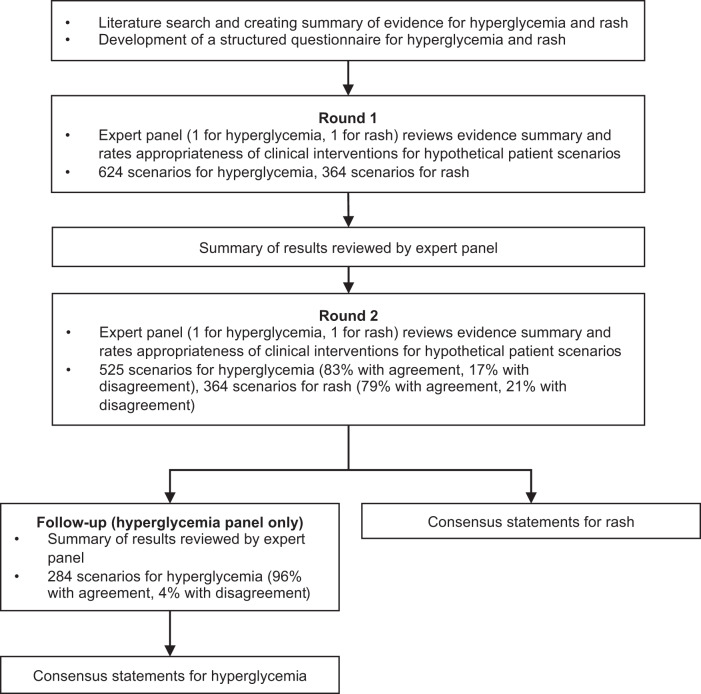
Delphi technique methodology.

The RAND Corporation/University of California Los Angeles (UCLA) modified Delphi panel method is a systematic and validated approach to collating individual opinions from experts and statistically generating areas of consensus, with the goal of creating a reliable expert consensus in the absence of definitive evidence^53,54^. In this study, two modified Delphi panels were conducted, one focusing on hyperglycemia and another on rash management in patients with HR+, HER2– ABC treated with alpelisib (Fig. 5).

### Expert panel

Ten experts each were recruited for the hyperglycemia and rash panels to represent a diverse range of backgrounds and expertize. Eligibility criteria for physicians included experience prescribing alpelisib (for medical oncologists/pharmacists) and managing hyperglycemia or rash; physicians would be familiar with the mechanism of action of alpelisib and how these AEs present in patients. Eligibility criteria for patient advocates included knowledge of clinical trials and guidelines for managing hyperglycemia and rash, knowledge of patient perspective regarding AE management, and ability to represent diversity in geography, demographic, and socioeconomic backgrounds in patients. The hyperglycemia panel was composed of 4 oncologists, 4 endocrinologists, 1 clinical pharmacist, and 1 patient advocate, whereas the rash panel was composed of 4 oncologists, 4 dermatologists, 1 clinical pharmacist, and 1 patient advocate. All physician experts and the clinical pharmacist in the hyperglycemia panel worked at an academic institution. In the rash panel, 5 of the physician experts worked at an academic institution and 3 worked at a mixed-practice setting (academic/community); the clinical pharmacist worked at a non-academic institution. All experts were based in the United States. No expert participated in more than one panel.

### Questionnaires and rating scale

An evidence summary (literature review) was developed for each panel based on recent reviews, clinical trials, case studies, guidelines, and real-world evidence obtained from targeted searches of the PubMed database on October 5, 2021, using the following search terms: “(alpelisib) AND (hyperglycemia)” and “(alpelisib) AND ((rash) OR (drug rash))”. The full search strategy is provided in Supplementary Table 1, and the list of publications reviewed is provided in Supplementary Table 2. For each panel, a structured questionnaire was developed based on the summary of current evidence in collaboration with panelists on the mechanism of action, risk factors, and management strategies for hyperglycemia and rash associated with alpelisib in patients with HR+, HER2– ABC.

The Delphi process involved 2 rounds per panel: in each round, experts were asked to review the evidence and rate the appropriateness of (in total) 1433 clinical interventions for hyperglycemia or 728 clinical interventions for rash on hypothetical patient scenarios using a scale of 1 (highly inappropriate, risks outweigh the benefits) to 9 (highly appropriate, benefits outweigh the risks). Aggregated first-round rating form results were reviewed at a virtual meeting. A moderator guided the discussion, focusing on items that generated the most disagreement, and ensured that feedback from all panelists was captured. Results from round 1 and the meeting discussion informed iterations needed for the round 2 rating form. No formal attempt to reach consensus was made during the meeting. Instead, at the conclusion of the meeting, experts completed the rating form a second time. Experts on the hyperglycemia panel decided some portions of the rating form needed to be revised and agreed to convene at a follow-up meeting to discuss their ratings of the revised sections.

### Data analysis

Data from the final rating form were then classified into 4 categories based on the median scores and dispersion. Disagreement was defined as being present if there were ≥2 ratings of 1–3 and ≥2 ratings of 7–9 for an intervention. The remaining categories were “appropriate,” defined as a median score of 7–9; “may or may not be appropriate,” defined as a median score of 4–6; and “inappropriate,” defined as a median score of 1–3; all without disagreement. For each panel, consensus statements and treatment algorithms were developed based on these ratings and were considered final after the review and approval of all panelists.

### Ethical approval and written informed consent

Modified Delphi panels do not involve human subjects as defined in the Code of Federal Regulations Title 45: Public Welfare, part 46 (45 CFR 46); thus institutional review board approval and written informed consent were not required.

### Reporting summary

Further information on research design is available in the Nature Research Reporting Summary linked to this article.

### Supplementary information

Supplementary Information

Reporting Summary

## Data Availability

The datasets generated during and/or analyzed during the current study are available from the corresponding author on reasonable request. Correspondence and requests for materials should be addressed to Emily J. Gallagher.

## Code availability

No custom code or mathematical algorithm was used in this study.
