# Supplementary material for: Managing hyperglycemia and rash associated with alpelisib: expert consensus recommendations using the Delphi technique
Source: NPJ Breast Cancer. 2024 Jan 31;10:12. doi: 10.1038/s41523-024-00613-x (PMC10831089; doi:10.1038/s41523-024-00613-x)
Supplement: Supplementary file 1 — Supplementary Information [file 41523_2024_613_MOESM1_ESM.pdf]

Supplementary Discussion Interactive plain language summary of “Managing Hyperglycemia and Rash Associated With Alpelisib: Expert Consensus Recommendations Using the Delphi Technique”

Managing Hyperglycemia and Rash Associated With Alpelisib: Expert Consensus Recommendations Using the Delphi Technique

Emily J. Gallagher[1,*]; Heather Moore[2]; Mario E. Lacouture[3]; Susan F. Dent[2]; Azeez Farooki[3,4]; Marcus D. Goncalves[4]; Claudine Isaacs[5]; Abigail Johnston[6]; Dejan Juric[7]; Zoe Quandt[8]; Laura Spring[7]; Brian Berman[9]; Melanie Decker[10]; Gabriel N. Hortobagyi[11]; Benjamin H. Kaffenberger[12]; Bernice Y. Kwong[13]; Timothy Pluard[14]; Ruta Rao[15]; Lee Schwartzberg[16]; Michael S. Broder[17]

[1]Division of Endocrinology, Diabetes and Bone Disease, Department of Medicine, and Tisch Cancer Institute, Icahn School of Medicine at Mount Sinai, New York, NY, USA; [2]Duke Cancer Institute, Duke University, Durham, NC, USA; [3]Department of Medicine, Memorial Sloan Kettering Cancer Center, New York, NY, USA; [4] Division of Endocrinology, Weill Department of Medicine, Weill Cornell Medicine, New York, NY, USA; [5]Lombardi Comprehensive Cancer Center, Georgetown University, Washington, DC, USA; [6]Surviving Breast Cancer, 305 Pink Pack, Miami, FL, USA; [7]Massachusetts General Hospital Cancer Center, Department of Medicine, Harvard Medical School, Boston, MA, USA; [8]School of Medicine, University of California, San Francisco, CA, USA; [9]University of Miami School of Medicine and Center for Clinical and Cosmetic Research, Aventura, FL, USA; [10]Woodland Memorial Hospital, Woodland, CA, and Kaiser Permanente, Sacramento, CA, USA; [11]Department of Breast Medical Oncology, The University of Texas MD Anderson Cancer Center, Houston, TX, USA; [12]Wexner Medical Center, The Ohio State University, Columbus, OH, USA; [13]Department of Dermatology, Stanford University School of Medicine, Stanford, CA, USA; [14]St. Luke's Hospital Koontz Center for Advanced Breast Cancer, Kansas City, MO, USA; [15]Rush Hematology, Oncology and Cell Therapy, Rush University Medical Center, Chicago, IL, USA; [16]West Cancer Center, Memphis, TN, USA; [17]PHAR, Beverly Hills, CA, USA.

*Corresponding author:
Emily J. Gallagher
Division of Endocrinology, Diabetes and Bone Disease
Department of Medicine
One Gustave L. Levy Place
Box 1055, Icahn School of Medicine at Mount Sinai
New York, NY 10029 USA

Email: Emily.Gallagher@mssm.edu

npj Breast Cancer

Managing Hyperglycemia and Rash Associated With Alpelisib: Expert Consensus Recommendations Using the Delphi Technique

What is alpelisib?

- **Alpelisib** is a medicine that is given in combination with **fulvestrant** (a type of **endocrine therapy**). It is used to treat **HR+, HER2**– breast cancer that has spread to other parts of the body and has a mutation or change in the DNA of the *PIK3CA* gene
- Alpelisib is given to people whose cancer got worse while on or after treatment with endocrine therapy
- Some people taking alpelisib may have an increase in blood sugar levels and may get a rash

Signs and symptoms of high blood sugar and rash

What is the purpose of this study?

- After the FDA approves a medicine, it can be taken by more people who are not part of a clinical study
- We looked at information from people treated in this setting
- We then used that information to make practical recommendations for managing common side effects experienced by people taking alpelisib

How was this study done?

In this study, we used the Delphi technique

- We assembled 2 groups of 10 experts each: 1 panel for high blood sugar, and 1 panel for rash

High blood sugar panel

4 endocrinologists
1 clinical pharmacist
4 oncologists
1 patient advocate

Rash panel

4 dermatologists
1 clinical pharmacist
4 oncologists
1 patient advocate

- There were 2 rounds of review for each panel. For each round, we asked experts to rate how appropriate different kinds of treatment and management strategies are for people who are taking alpelisib and experiencing high blood sugar or rash
- We combined and analyzed their response scores to identify expert consensus recommendations

Delphi panel recommendations: Prevention, monitoring, and management*

High Blood Sugar

How can high blood sugar be prevented while taking alpelisib?

- Low carbohydrate diet (60-130 g of carbohydrates per day) is the preferred diet for people who are going to start taking alpelisib
- Your doctor may recommend that you follow a ketogenic diet (high fat and very low carbohydrate diet, with <50 g of carbohydrates per day; this type of diet is still being studied) and/or fasting before starting alpelisib
- Your doctor may ask you to take a medication called metformin to prevent your blood sugar from going up

How is blood sugar tested?

Get a fasting blood sample (No food or drink other than water for 8-12 hours before the test)

You can use a glucose monitor at home

How often should blood sugar be checked?

1x/week	2x/week	Daily
Most People	People who have obesity and prediabetes	People who are ≥70 years old, have obesity and prediabetes

High blood sugar definition, prevention, and medications used

Management by blood sugar level

Rash

How can rash be prevented while taking alpelisib?

Your doctor may prescribe **nonsedating** or **non-drowsy H1 antihistamines** before you start taking alpelisib — Examples are cetirizine and loratadine

How is rash treated?

Doctors use two types of medicines to start treating rash:

- **Nonsedating or non-drowsy H1 antihistamines**
- **Topical steroids**

If you develop swelling in the deeper layers of the skin (known as angioedema), your doctor will ask you to:

- Temporarily stop taking alpelisib and start taking steroid tablets, or
- Permanently stop taking alpelisib

Rash management

*These recommendations are meant to be a guide, and your doctor may manage your condition differently depending on individual circumstances.

Glossary

Managing Hyperglycemia and Rash Associated With Alpelisib: Expert Consensus Recommendations Using the Delphi Technique

1. High blood sugar and rash observed in people taking alpelisib

High blood sugar and **rash** are 2 common side effects experienced by people taking alpelisib. **However, not everyone taking alpelisib will experience high blood sugar or rash, and this <u>does not</u> mean that the drug is not working**.
Half of people who experienced **high blood sugar** or **rash** first experienced them within[1]:

15 days
for high blood sugar

12 days
for rash

What are the signs and symptoms of high blood sugar?

- Increased appetite with weight loss
- Excessive thirst
- More frequent urination or a higher amount of urine than normal
- Dry mouth
- Dry or flushed skin
- Vomiting
- Nausea
- Fruity odor on breath
- Confusion

What does rash look like in people taking alpelisib?

- Usually small (<1 cm), red, flat, and raised bumps on the skin
- Usually found on the torso, arms, and legs
- May be accompanied by itching or a burning sensation

The picture below shows what rash looks like in a person taking alpelisib.[2]

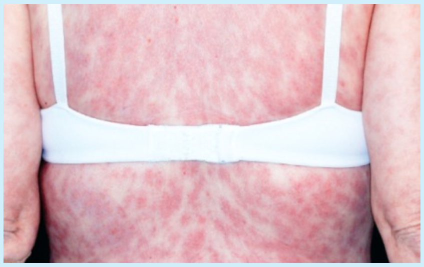

Reprinted from Rugo HS, et al. A multidisciplinary approach to optimizing care of patients treated with alpelisib. *Breast*. 2022;61:156-167.[2] https://doi.org/10.1016/j.breast.2021.12.016
© 2022 Rugo HS, et al. https://creativecommons.org/licenses/by/4.0/

These recommendations are meant to be a guide, and your doctor may manage your condition differently depending on individual circumstances.

Glossary

2. Defining blood sugar status at baseline, preventing high blood sugar, and preferred medicines to manage high blood sugar

Your doctor may ask you to take a blood test called **HbA1c** before you start alpelisib (baseline). This lets your doctor check how much sugar you have had in your blood over the past 3 months. Your blood sugar levels will tell your doctor if you have prediabetes or diabetes:

HbA1c levels[3]	Below 5.7%	5.7% to 6.4%	6.5% or above
	Normal	Prediabetes	Diabetes

Delphi panel recommendations

Do I need to see an endocrinologist before I start taking alpelisib?

- ✗ If you **do not have obesity** and have normal **HbA1c**: No, your doctor does not need to refer you to an **endocrinologist**
- ✓ If you have **type 2 diabetes mellitus**, or
- ✓ If you are 70 years old or older, **have obesity** and **prediabetes**

  Yes, your doctor will likely refer you to an **endocrinologist** for evaluation before starting alpelisib
- ? If your **HbA1c** is 8% or higher, your doctor may need further evaluation to see if you can take alpelisib

Does everyone taking alpelisib need to take metformin to prevent high blood sugar?

Your doctor may ask you to take metformin before you start alpelisib therapy if, based on your **HbA1c**, your blood sugar levels are in the range of:

Normal or **Prediabetes**

How is high blood sugar treated if it develops while taking alpelisib?

Your doctor may ask you to take the following types of medicines to control high blood sugar while you are on alpelisib:

#1 **Metformin** is the preferred first medicine that will usually be prescribed by your doctor

#2-3 **Sodium-glucose cotransporter-2 (SGLT2) inhibitors, thiazolidinediones, or glucagon-like peptide-1 (GLP-1) receptor agonists** may be prescribed as an addition to metformin (or as an alternative to metformin if you cannot take metformin)

#3 **Dipeptidyl-peptidase 4 (DPP4) inhibitors** may also be prescribed by your doctor if your blood sugar is still not controlled by metformin and a second drug

! **Insulin** may be prescribed by your doctor in certain instances. However, **insulin** should not be used as the first or second drug to treat high blood sugar while on alpelisib

Management by blood sugar level

These recommendations are meant to be a guide, and your doctor may manage your condition differently depending on individual circumstances.

Glossary

3. Managing high blood sugar while taking alpelisib: Delphi panel recommendations

Your doctor will first check how high your fasting blood sugar is:

<100 mg/dL	100-160 mg/dL	>160-250 mg/dL	>250-500 mg/dL	>500 mg/dL
Normal	Moderately high	High	Very high	Extremely high

Having high blood sugar levels while taking alpelisib does not necessarily mean you have diabetes. Taking alpelisib may cause blood sugar levels to go up in some people, but this is reversible.

My doctor says I have high blood sugar, what's next?

The chart below summarizes expert recommendations for managing high blood sugar in people taking alpelisib.

Depending on your fasting blood sugar level, your doctor may

Fasting blood sugar levels	Prescribe metformin, or ask you to continue taking it or increase its dose	Prescribe another medicine in addition to metformin	Refer you to an **endocrinologist**	Ask you to temporarily stop taking alpelisib
Moderately high	✓			
High	✓ or	✓		
Very high	✓	✓	✓	✓
Extremely high	✓	✓	✓	✓

Your blood sugar may go higher than normal for a second time. If it does , and depending on how high it is, your doctor may:

- Prescribe **insulin** (but not as the first or second drug)
- Ask you to delay or stop taking alpelisib

These recommendations are meant to be a guide, and your doctor may manage your condition differently depending on individual circumstances.

Glossary

4. Managing rash while taking alpelisib: Delphi panel recommendations

Your doctor will check to see how much of your body is covered by the rash. Your doctor will then determine the percentage of body surface area (BSA) affected:

% BSA affected	Below 10%	10% to 30%	More than 30%
	Mild	Moderate	Severe

My doctor assessed my rash and determined the % BSA affected, what's next?

The chart below summarizes the recommendations from experts for managing rash in people taking alpelisib. Depending on the % BSA affected by the rash, your doctor may recommend you to a dermatologist to determine if the rash is caused by alpelisib.

% BSA affected	Your doctor may: Prescribe or increase the dose of antihistamine and prescribe topical steroids	Prescribe steroids that are taken orally	Refer you to a dermatologist or allergist	Ask you to temporarily stop taking alpelisib
Mild	✓			
Moderate	✓	✓	✓	✓
Severe	✓	✓	✓	✓

If your rash does not improve after **1-2 weeks**, your doctor may lower your dose of alpelisib or ask you to permanently stop taking alpelisib.

What kinds of antihistamines are used for managing rash in people taking alpelisib?

Depending on how your rash is responding to treatment, your doctor may tell you to use one or more of the following:

- #1 **Nonsedating or non-drowsy H1 antihistamines**; your doctor may tell you to increase the dose depending on your response
- #2 Add **sedating or drowsy H1 antihistamine**
- #3 Add **H2 antihistamine**

Managing Hyperglycemia and Rash Associated With Alpelisib: Expert Consensus Recommendations Using the Delphi Technique

Glossary

Angioedema: Swelling in the deeper layers of the skin, frequently observed in the eyelids, mouth or genitals, usually as a reaction to a medicine or something to which you are allergic. Many people will also have urticaria or hives, which is a rash that is raised and itchy

Antihistamine: A type of medicine that stops the effect of histamines, a chemical released by the body as a response to a foreign substance (such as a drug), which can cause allergy symptoms such as rashes and itchy skin

Dipeptidyl-peptidase 4 (DPP4) inhibitors: A class of medicines used to treat high blood sugar. Examples include alogliptin, linagliptin, and sitagliptin

Endocrine therapy: A class of medicines that adds, blocks, or removes hormones. These medicines are used to stop or slow the growth of certain subtypes of breast cancer that depend on these hormones to grow and spread

Endocrinologist: A doctor who specializes in treating hormone-related medical conditions, including diabetes mellitus

Fulvestrant: A type of medicine classified as a hormone or **endocrine therapy** that is used to treat breast cancer

Glucagon-like peptide-1 (GLP-1) receptor agonists: A class of medicines used to treat high blood sugar. Examples include dulaglutide, exenatide, liraglutide. and semaglutide

H1 antihistamines: Medicines that stop the effect of histamines by blocking their receptors called H1 receptors. Some H1 antihistamines can cause a person to become drowsy or sleepy (**drowsy or sedating antihistamines**, including diphenhydramine and hydroxyzine), while some do not (**non-drowsy or nonsedating antihistamines**, including cetirizine, fexofenadine, levocetirizine, and loratadine)

H2 antihistamines: Medicines that stop the effect of histamines by blocking their receptors called H2 receptors. Examples of H2 antihistamines include cimetidine, famotidine, and nizatidine

HR+, HER2– breast cancer: A subtype of breast cancer wherein the cancer has hormone receptors (HRs) inside the cells, but the cells do not have a protein called human epidermal growth factor receptor 2 (HER2)

Insulin: A type of hormone that is normally produced by the pancreas and functions to control and lower blood sugar levels. Insulin is also available as a medicine for certain people that require it

Ketogenic diet: A type of diet that is very low in carbohydrates (<50 g/day), moderate in protein, and high in fat

Obese: Weight that is considered high for a given height, determined by calculating a person's body mass index (BMI). The BMI is calculated by dividing a person's weight in kilograms by the square of the person's height in meters (BMI = kg/m^2). A BMI of 30 kg/m^2 or higher is considered obese

Sodium-glucose cotransporter-2 (SGLT2) inhibitors: A class of medicines used to treat high blood sugar. Examples include canagliflozin, dapagliflozin, empagliflozin, and ertugliflozin

Thiazolidinediones: A class of medicines used to treat high blood sugar. Examples include pioglitazone and rosiglitazone

Topical steroid: A type of medicine that is applied to the skin to reduce inflammation and irritation. Examples include triamcinolone acetonide 0.5% and betamethasone dipropionate 0.05%

Type 2 diabetes mellitus: : A medical condition in which either the body is not producing enough insulin or the cells are unable to effectively respond to or use insulin, or both. This leads to high blood glucose levels

References

1. Piqray [prescribing information]. East Hanover, NJ: Novartis Pharmaceuticals Corporation.
2. Rugo HS, et al. A multidisciplinary approach to optimizing care of patients treated with alpelisib. *Breast*. 2022;61:156-167.
3. American Diabetes Association Professional Practice Committee. Standards of Medical Care in Diabetes—2022. *Diabetes Care*. 2022;45(suppl.1):S254-S255.

Acknowledgments

The authors thank Mary Lou Smith, JD, MBA, Research Advocacy Network, for her contributions to this plain language summary. Medical editorial assistance was provided by Audrey Clement So, MD, from Healthcare Consultancy Group, LLC, and was funded by Novartis Pharmaceuticals Corporation.

Supplementary Table 1 Search strategy for identifying articles[a] for the evidence summary for hyperglycemia and rash

Hyperglycemia	Rash
• **Search 1.** alpelisib: 256 hits<br>• **Search 2.** (alpelisib) AND (breast cancer): 147 hits<br>• **Search 3.** (alpelisib) AND ((rash) OR (drug rash)): 22 hits<br>• **Search 4.** (alpelisib) AND (hyperglycemia): 31 hits<br>• **Search 5.** (drug rash) AND (management OR treatment) AND ("medication-induced" OR "drug-induced") AND ((y_5[Filter]) AND (review[Filter])): 97 hits<br>• **Search 6.** (hyperglycemia) AND (management OR treatment) AND ("medication-induced" OR "drug-induced") AND ((y_5[Filter]) AND (review[Filter])): 12 hits	• **Search 1.** alpelisib: 256 hits<br>• **Search 2.** (alpelisib) AND (breast cancer): 147 hits<br>• **Search 3.** (alpelisib) AND ((rash) OR (drug rash)): 22 hits<br>• **Search 4.** (drug rash) AND (management OR treatment) AND ("medication-induced" OR "drug-induced") AND ((y_5[Filter]) AND (review[Filter])): 97 hits

[a]Articles were initially chosen for relevance based first on the title and then based on abstract screening. Full articles remaining after those 2 screens were further reviewed and the most relevant ones were chosen based on the topic.

Supplementary Table 2 Publications included in the evidence summary for the hyperglycemia and rash panels

Hyperglycemia panel	Rash panel
1. Shields M, et al. *Oncotarget*. 2020;11:3793-3799.	1. Shields M, et al. *Oncotarget*. 2020;11:3793-3799.
2. Piqray [prescribing information]. East Hanover, NJ: Novartis Pharmaceuticals Corp.	2. Piqray [prescribing information]. East Hanover, NJ: Novartis Pharmaceuticals Corp.
3. National Institutes of Health; National Cancer Institute. Surveillance, epidemiology, and end results program. Cancer stat facts: female breast cancer. https://seer.cancer.gov/statfacts/html/breast.html via the Internet. Accessed October 27, 2021.	3. National Institutes of Health; National Cancer Institute. Surveillance, epidemiology, and end results program. Cancer stat facts: female breast cancer. https://seer.cancer.gov/statfacts/html/breast.html via the Internet. Accessed October 27, 2021.
4. Waks AG, Winer EP. *JAMA*. 2019;321:288-300.	4. Waks AG, Winer EP. *JAMA*. 2019;321:288-300.
5. Pan H, et al. *N Engl J Med*. 2017;377:1836-1846.	5. Pan H, et al. *N Engl J Med*. 2017;377:1836-1846.
6. Mosele F, et al. *Ann Oncol*. 2020;31:377-386.	6. Mosele F, et al. *Ann Oncol*. 2020;31:377-386.
7. Miller TW, et al. *Breast Cancer Res*. 2011;13:224.	7. Miller TW, et al. *Breast Cancer Res*. 2011;13:224.
8. Sobhani N, et al. *J Cell Biochem*. 2018;119:4287-4292.	8. Sobhani N, et al. *J Cell Biochem*. 2018;119:4287-4292.
9. Toppmeyer DL, Press MF. *Cancer Med*. 2020;9:6463-6472.	9. Toppmeyer DL, Press MF. *Cancer Med*. 2020;9:6463-6472.
10. André F, et al. *N Engl J Med*. 2019;380:1929-1940.	10. André F, et al. *N Engl J Med*. 2019;380:1929-1940.
11. Rugo HS, et al. *Ann Oncol*. 2020;31:1001-1010.	11. Rugo HS, et al. *Ann Oncol*. 2020;31:1001-1010.
12. Ferdinand KC, Nasser SA. *Curr Med Res Opin*. 2015;31:913-923.	12. Mayer IA, et al. *Clin Cancer Res*. 2017;23:26-34.
13. Goncalves MD, et al. *N Engl J Med*. 2018;379:2052-2062.	13. Ando Y, et al. *Cancer Sci*. 2019;110:1021-1031.
14. Hopkins BD, et al. *Nature*. 2018;560:499-503.	14. Rugo HS, et al. *Lancet Oncol*. 2021;22:489-498.
15. Baselga J, et al. *Lancet Oncol*. 2017;18:904-916.	15. Majeed U, et al. *Front Oncol*. 2021;11:726785.
16. Di Leo A, et al. *Lancet Oncol*. 2018;19:87-100.	16. Mayer IA, et al. *Clin Cancer Res*. 2019;25:2975-2987.
17. Nguyen P, et al. *Cureus*. 2021;13:e14796.	17. Wang DG, et al. *Breast Cancer Res Treat*. 2020;183:227-237.
18. Carrillo M, et al. *AACE Clin Case Rep*. 2021;7:127-131.	18. Teng Y, et al. *Cells*. 2021;10:1219.
19. Farah SJ, et al. *AACE Clin Case Rep*. 2020;6:e349-e351.	19. Bruce I, et al. *Bioorg Med Chem Lett*. 2012;22:5445-5450.

20. Blow T, et al. *Integr Cancer Ther*. 2021;20:15347354211032283.	20. Bendell JC, et al. *J Clin Oncol*. 2012;30:282-290.
21. Sahakian N, et al. *Clin Diabetes Endocrinol*. 2021;7:17.	21. Baselga J, et al. *Lancet Oncol*. 2017;18:904-916.
22. Goldman JW, et al. *Oncologist*. 2016;21:1326-1336.	22. Sharman JP, et al. *J Clin Oncol*. 2019;37:1391-1402.
23. Busaidy NL, et al. *J Clin Oncol*. 2012;30:2919-2928.	23. Patnaik A, et al. *Ann Oncol*. 2016;27:1928-1940.
24. Cheung YM, et al. *Curr Probl Cancer*. 2021;100776.	24. Sheu J, et al. *Clin Breast Cancer*. 2015;15:e77-81.
25. Villadolid J, et al. *Transl Lung Cancer Res*. 2015;4:576-583.	25. Pan RY, et al. *Clin Pharmacol Ther*. 2017;102:86-97.
26. Lew S, Chamberlain RS. *Anticancer Res*. 2016;36:1711-1718.	26. Chen CB, et al. *J Immunol Res*. 2018;6431694.
27. Saglio G, et al. *N Engl J Med*. 2010;362:2251-2259.	27. Gerogianni K, et al. *Mol Diagn Ther*. 2018;22:297-314.
28. Shah RR. *Drug Saf*. 2017;40:211-228.	28. Balakirski G, Merk HF. *Cutan Ocul Toxicol*. 2017;36:307-316.
29. Caffa I, et al. *Nature*. 2020;583:620-624.	
30. Crouthamel MC, et al. *Clin Cancer Res*. 2009;15: 217-225.	
